# The Mississippi Delta Cardiovascular Health Examination Survey: Study Design and Methods

**DOI:** 10.1155/2014/861461

**Published:** 2014-01-01

**Authors:** Vanessa L. Short, Tameka Ivory-Walls, Larry Smith, Fleetwood Loustalot

**Affiliations:** 1 Mississippi State Department of Health, 570 East Woodrow Wilson, Osborne-429B, Jackson, MS 39215, USA; 2 Office of Preventive Health, Mississippi State Department of Health, 522 West Park Avenue, Suite P, Greenwood, MS 38930, USA; 3 Office of Preventive Health, Mississippi State Department of Health, 570 East Woodrow Wilson, Osborne-429B, Jackson, MS 39215, USA; 4 Division for Heart Disease and Stroke Prevention, Centers for Disease Control and Prevention, 1600 Clifton Road, Atlanta, GA 30333, USA

## Abstract

Assessment of cardiovascular disease (CVD) morbidity and mortality in subnational areas is limited. A model for regional CVD surveillance is needed, particularly among vulnerable populations underrepresented in current monitoring systems. The Mississippi Delta Cardiovascular Health Examination Survey (CHES) is a population-based, cross-sectional study on a representative sample of adults living in the 18-county Mississippi Delta region, a rural, impoverished area with high rates of poor health outcomes and marked health disparities. The primary objectives of Delta CHES are to (1) determine the prevalence and distribution of CVD and CVD risk factors using self-reported and directly measured health metrics and (2) to assess environmental perceptions and existing policies that support or deter healthy choices. An address-based sampling frame is used for household enumeration and participant recruitment and an in-home data collection model is used to collect survey data, anthropometric measures, and blood samples from participants. Data from all sources will be merged into one analytic dataset and sample weights developed to ensure data are representative of the Mississippi Delta region adult population. Information gathered will be used to assess the burden of CVD and guide the development, implementation, and evaluation of cardiovascular health promotion and risk factor control strategies.

## 1. Introduction

Cardiovascular disease (CVD) is the leading cause of mortality in the United States [1], accounting for over 25% of all deaths [1]. CVD data collected by surveillance systems allow for the appropriate allocation of limited resources and focused programmatic planning and intervention. National surveillance systems track CVD and its related risk factors using a collection of representative surveys (e.g., National Health and Nutrition Examination Survey (NHANES)). However, equivalent regional or local level surveillance of CVD is not currently available, and coordination of CVD surveillance is lacking [2]. Development of a replicable, scalable, and flexible model for local level surveillance is needed, particularly among vulnerable populations potentially underrepresented in current systems.

The 18-county Mississippi Delta region consistently ranks among the most disadvantaged areas in the nation, with approximately one-quarter of its population living below the federal poverty level (Table 1) [3]. Residents of the Mississippi Delta region experience age-adjusted rates of death due to major CVDs that are considerably higher than Mississippi and national rates and marked racial health disparities exist (Table 2).

States and localities benefit from reliable, timely, and accurate health information. The Behavioral Risk Factor Surveillance System (BRFSS) is frequently used to track a broad range of health indicators and direct programmatic planning at the state level. The methodology of the BRFSS allows for a large annual sample size and comparisons across states and large metropolitan areas. However, the BRFSS has limitations, including the use of random-digit-dialing to select participants and reliance on self-reported data. Moreover, telephone-based surveillance systems have noncoverage biases and comparisons between self-reported and measured health metrics have found inconsistencies, with significant over- and underestimation of CVD risk factors among population subgroups [5–9].

To further develop a model for CVD surveillance at the regional level and to examine the CVD risk factors in an area with significant health needs, the Mississippi State Department of Health (MSDH), with support from the Centers for Disease Control and Prevention (CDC), developed and initiated a Cardiovascular Health Examination Survey (CHES) in the Mississippi Delta region. The Delta CHES will provide a baseline examination of CVD and related risk factors, which can be later replicated to assess progression and/or progress among adult residents of the Mississippi Delta region. The primary objectives of Delta CHES are (1) to determine the prevalence and distribution of CVD and CVD risk factors using self-reported and directly measured health metrics and (2) to assess environmental perceptions and existing policies that support or deter healthy choices. The secondary objectives are (1) to develop a replicable, regional-level data collection model for use in future studies and (2) to create a blood repository for use in future ancillary studies. Information gathered will help guide the state in the development, implementation, and evaluation of cardiovascular health promotion and risk factor control strategies. Here we describe the Delta CHES design and methods.

## 2. Materials and Methods

### 2.1. Development of the Model

The methods and model used in the Delta CHES were informed using comparable pilot surveys conducted in four US states (Washington [9], Arkansas [10], Kansas, and Oklahoma). Previous pilot surveys were undertaken to address a gap in available data at the local, regional, and state levels. The Delta CHES model was developed to provide regional estimates by using a detailed process and outcome evaluation conducted among the four pilot states and incorporating historical accounts of other local and regional data collection surveys (e.g., Survey of the Health of Wisconsin [11]; New York City Health and Nutrition Examination Survey [12]).

Lessons learned from previous pilot surveys influenced the ordering of tasks during planning stages, the selection of a sampling method, and the choice for the Delta CHES model. Potential causes of delay identified by pilot surveys, such as institutional review board (IRB) approval, staffing, questionnaire development, and selection and use of contractors for data collection and management were prioritized during the initial phases of Delta CHES. Methods employed by prior examination surveys varied from in-home data collection for all aspects of the survey to telephone-based data collection of self-reported data and measured health data conducted at clinic sites. Variations in response rates and health outcome findings from prior surveys led to the selection of in-home data collection of self-reported and measured data for Delta CHES.

### 2.2. Study Design

Delta CHES is a population-based, cross-sectional study. Survey data, anthropometric measures, and blood samples are collected from individuals ≥18 years from the adult, noninstitutionalized population of the Mississippi Delta region. The MSDH IRB approved all Delta CHES procedures and documents. The planning phase began in 2011, and the implementation phase began in late 2012 with expectations of completion in early 2014.

### 2.3. Study Population

**Figure 1:**
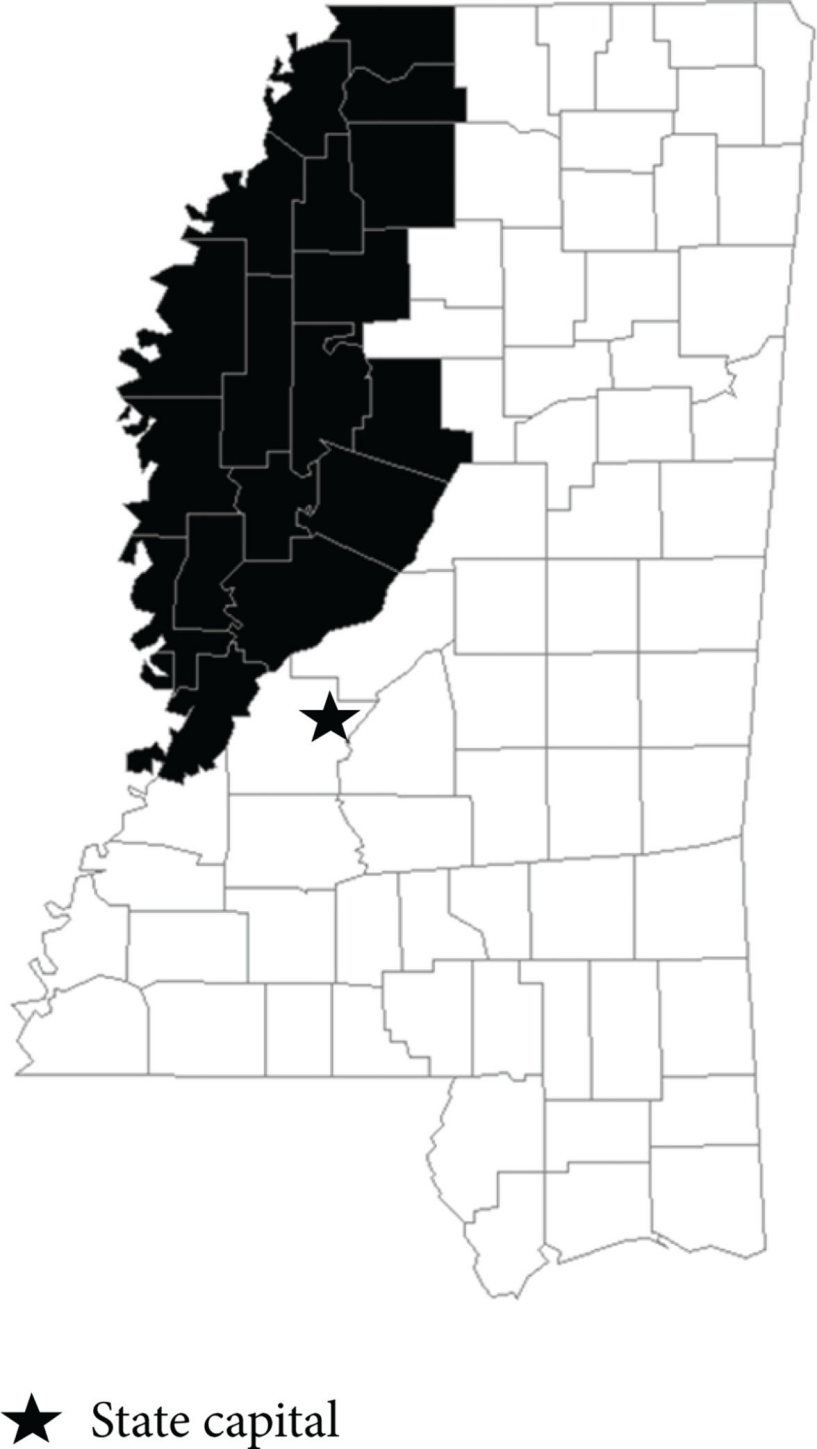
The 18-county Mississippi Delta region. The Mississippi Delta region (shaded in black) includes the following counties: Bolivar, Carroll, Coahoma, Desoto, Holmes, Humphreys, Issaquena, Leflore, Panola, Quitman, Sharkey, Sunflower, Tallahatchie, Tate, Tunica, Warren, Washington, and Yazoo.

The Mississippi Delta region, an area the size of Connecticut in the Northwest part of Mississippi with a population of approximately 553,000, includes the following 18 counties: Bolivar, Carroll, Coahoma, Desoto, Holmes, Humphreys, Issaquena, Leflore, Panola, Quit-man, Sharkey, Sunflower, Tallahatchie, Tate, Tunica, Warren, Washington, and Yazoo (Figure 1). Individuals are eligible to participate in Delta CHES if they are ≥18 years of age, nonpregnant, reside within the 18-county Mississippi Delta region, and are able to sign an informed consent form. Persons who cannot legally sign a consent form, persons with psychiatric, cognitive, or developmental disorders, persons with hemophilia, those undergoing treatment for cancer, non-English speakers, and those who are not expected to be in residence within 30 days of selection are excluded.

### 2.4. Sample Size and Power

One of our main interests is to compare prevalence estimates between non-Hispanic blacks and non-Hispanic whites, within the limits of sample size feasibility. Table 3 shows the sample sizes needed to have a power of 80% to detect a difference in leading CVD risk factor (i.e., dyslipidemia, hypertension, and, diabetes) prevalence between these two groups (Table 3). Based on a similar study conducted in Arkansas [10], we expect about one-third of contacted houses to have an eligible adult willing to participate and a 65% completion rate among surveys started. With 80% probability of detecting a true difference, at the 0.05 significance level, between equal-sized samples of non-Hispanic blacks and non-Hispanic whites, we estimate a total sample size of approximately 1,000 which will allow us to detect differences in selected CVD risk factors.

### 2.5. Sampling and Recruitment

The Mississippi Delta region can be a difficult place to conduct health surveys due to many factors, including the rurality of the area, low landline telephone coverage, and low socioeconomic status of many residents. Therefore, innovative means are used to sample and recruit participants into Delta CHES. First, a two-stage address-based sampling method is used to sample households and participants. This approach reduces the potential coverage bias of traditional random-digit dialing. In the latter half of 2012, approximately 38% of US homes had only cellular phones [13]. In a recent comparison with other states, Mississippi had one of the highest proportions of adults in wireless-only households (42.3%) [14]. Further, adults living in cellular phone-only households tend to be younger, have lower incomes, and be members of minority populations [14], increasing the risk for coverage bias in Mississippi Delta communities with high percentages of non-Hispanic blacks and lower income households.

The frame for the first stage of sampling, which consisted of residential addresses in the Delta region, was purchased from a private vendor (Marketing Systems Group; http://www.m-s-g.com). The basis of this file is the US Postal Service Computerized Delivery Sequence File, which contains nearly all delivery-point addresses served by the US Postal Service [15]. Geographic information systems technology was used to construct an address frame that matched the geographies of the Delta CHES population and addresses were matched to telephone numbers (~47% matching rate). Approximately 6,000 households were randomly selected from the list of addresses.

The second stage of sampling involves the selection of eligible adults for participation in Delta CHES. Each randomly selected household is mailed a letter which describes the study, identifies the household as being part of the survey sample, and informs residents that field staff would be calling the household. The letter also requests the household to contact study staff via a toll-free number to confirm the telephone number on file or to provide a telephone number so that they can be contacted regarding participation. This letter is particularly useful for those households where telephone matching is unsuccessful. Any returned or undeliverable letters are receipted into an electronic management system and the outcome of the mailing logged with an appropriate disposition code.

For addresses with an associated telephone number, trained telephone interviewers call the households to complete an enumeration survey. Interviewers make up to seven attempts to contact an adult household member at different times of the day and different days of the week including weekends. If contact is made, interviewers verify that they have reached the correct household, introduce and briefly explain Delta CHES, and administer the enumeration survey using a computer-assisted telephone interviewing (CATI) system. The name, age, sex, and phone number of each eligible household member is collected. Each contact attempt is tracked and households where telephone contact attempts are unsuccessful are reassigned for in-person enumeration.

An in-person household enumeration is used for any household for which there is not a valid telephone number or when telephone contact attempts are unsuccessful. Due to the high travel costs associated with in-person enumeration, only one visit is made to each home. The enumeration survey is programmed on handheld devices and allows for direct entry of information (i.e., name, age, sex, and phone number of each eligible household member) by interviewers. A door hanger is left at those houses where field staff are unable to reach a person during the visit. The door hanger describes Delta CHES and requests the household to call study staff via a toll-free telephone number to discuss participation in Delta CHES.

Once households are enumerated, one to two (depending on household size) eligible adults from each household are selected. Sampled residents are called to obtain verbal agreement to participate in Delta CHES. Up to seven attempts are made to reach each selected potential participant at different times of the day and different days of the week and a CATI recruitment script and data collection instrument is used to electronically capture all responses. If the randomly selected adult agrees to participate in Delta CHES, field-screening staff verify contact information and send this information to the data collection staff. At this time, each participant is assigned a unique identification number which is used throughout the study for tracking purposes.

To compensate for recruitment challenges of the random sampling strategy, recruitment of participants also includes a “self-selected” sample. Similar strategies have been used by other major CVD epidemiological studies, including the Framingham Heart Study [16] and the Jackson Heart Study [17]. Enrollment into Delta CHES is open to volunteers who meet census-derived age, sex, and race eligibility criteria for the 18-county region. An enrollment algorithm based on US Census data for each county determines anticipated recruitment for each age*sex*race subgroup. Individuals interested in participating in Delta CHES are asked to call the study’s toll-free number to complete the screener questionnaire. The name and contact information of each recruited participant is subsequently sent to the data collection staff. The volunteer sample is targeted to not exceed the sample size of those randomly selected and at the end of the study, randomly and self-selected participants will be compared to assess differences in key characteristics.

### 2.6. Data Collection

Examination Management Services, Inc. (EMSI), a provider of specimen collection services for clinical trials and epidemiological studies, is responsible for scheduling and completing an interview and examination appointment at each participant’s home. All field workers are required to complete training on the Delta CHES data collection protocol and adherence to National Institutes of Health data collection standards and protection of human subjects and obtain EMSI Human Participation and Protection certification prior to making any study visits. EMSI calls each participant 48 hours prior to the study visit to request a 9-hour fast. At the study home visit, written consent is obtained and questionnaire data, anthropometric measurements and blood samples are collected. The main Delta CHES questionnaire domains, physical measures, and blood tests are found in Table 4. A label containing the participant’s unique identification number is placed on all study-related materials at each data collection visit.

#### 2.6.1. Questionnaire

The Delta CHES questionnaire consists of up to 281 questions (depending on skip patterns) and is interviewer-administered with responses recorded directly on the paper form. The questionnaire includes questions related to medical history, CVD risk factors and behaviors (e.g., smoking, alcohol use, physical activity, and diet), and sociodemographics. Physical and built environments and community perceptions and existing health-related policies that support or deter healthy choices are also assessed. Participants are asked to present prescription and nonprescription medicines, including vitamins, supplements, and over-the-counter medications. The draft questionnaire was field-tested at a MSDH office in the Mississippi Delta region with community volunteers. Reactions, difficulties, and questions by respondents were noted systematically and discussed by Delta CHES staff and the questionnaire was revised accordingly. Many of the survey questions originated from established national (i.e., NHANES) or state-based surveys (e.g., BRFSS). The complete Delta CHES questionnaire is available on the study’s website [18].

#### 2.6.2. Physical Examination

The physical examination includes anthropometric and blood pressure measurements. Participants are measured for height, weight, and hip and waist circumference. Weight is measured on hard surface floor (if available) using a digital scale (Healthometer) and recorded in pounds to the nearest decimal. Height is measured on hard surface floor (if available) using a metal tape measure with the participant’s heels and buttocks against a wall and recorded in feet and inches to the nearest half inch. Waist circumference is measured with a soft measuring tape at umbilicus level, keeping the tape parallel to the floor. The measurement is recorded in inches to the nearest half inch. Hip circumference is measured at the widest point of the participant’s buttocks with the soft measuring tape parallel to the floor. It is recorded in inches to the nearest half inch. Resting blood pressure is measured in the right arm, if available, three times in the seated position with an American Diagnostic Corporation (Hauppauge, New York) sphygmomanometer. There is a five-minute waiting period before taking the first blood pressure and a 30-second period between measurements. Equipment is calibrated daily using standardized protocols and compared to the previous day’s measurement.

#### 2.6.3. Biological Specimen Collection

After administering the questionnaire and taking anthropometric measurements, interviewers collect approximately 36mL of blood from each participant in a requested fasting state. All specimens are processed and shipped overnight to laboratories for analysis according to protocol (Table 5). The Mississippi Public Health Laboratory (Jackson, MS), a laboratory currently participating in the CDC Lipid Standardization Program [19], is responsible for the lipid assay. All remaining samples are analyzed by Laboratory Corporation of America (LabCorp) [20].

A portion of the blood collected during examination is stored for potential use in future health studies. Participants who provide consent for storage of blood have 5mL of their serum stored at −70°C in a secure laboratory space at the Mississippi Public Health Laboratory. Future studies may be conducted using stored samples which will be identified using the participant’s unique identification number. Researchers from public health agencies, universities, and other scientific centers can submit proposals to use the stored specimens, as well as other Delta CHES data. All research proposals will be reviewed for scientific merit and integrity by the MSDH IRB.

#### 2.6.4. Food Frequency Questionnaire

At the end of the study visit, each participant is provided a food frequency questionnaire (FFQ), one-page instructions for completing the FFQ, a pencil, and a postage-paid preaddressed return envelope. The Delta CHES toll-free 800 number is included in the instructions and participants can call study staff with questions concerning the FFQ or to obtain assistance with completing it. The FFQ was developed by the Nutrition Assessment Shared Resource of Fred Hutchinson Cancer Research Center (Seattle, WA). This FFQ was chosen because it has an advantage of being comparable to the other CHES exam sites (i.e., Arkansas) and can provide an overall assessment of sodium intake, an important CVD risk factor. The self-administered FFQ booklet asks participants to report the frequency of consumption and portion size of approximately 120 line items over the past three months. Each line item is defined by a series of foods or beverages. Participants complete the FFQ and return it to MSDH. Approximately two weeks after the initial study visit, a call is made to nonresponding participants to remind them to complete the FFQ. Two weeks after the reminder call, a second call is made to nonresponding participants.

#### 2.6.5. Pedometer and Daily Steps Diary

Following completion of the main Delta CHES data collection, participants are also given a pedometer kit to self-monitor physical activity for five consecutive days. Pedometers are a simple, inexpensive way to measure physical activity [21] and five consecutive days of data collection are enough to achieve a reliable and valid estimate of physical activity [22]. Kits include a pedometer, instructions on how to use the pedometer, a recording diary, and a postage-paid preaddressed return envelope. Interviewers provide assistance in opening and resetting the pedometers, if necessary. Study pedometers are the Yamax SW200 models, which demonstrate high concordance with accelerometers under laboratory conditions and in field settings [23]. The Yamax SW200 has been recommended in a number of validation studies as a preferred model for measuring daily steps in free-living populations [24, 25]. Participants are instructed to reset their pedometer to zero each morning, go about their typical activities, remove the pedometer only while bathing, showering, or swimming, and record their day-end steps taken on the provided diary. The completed diary is returned to MSDH; participants keep the pedometers. Approximately two weeks after the initial study visit, a call is made to nonresponding participants to remind them to complete the pedometer diary. Two weeks after the reminder call, a second call is made to nonresponding participants.

### 2.7. Data Integrity and Quality

Several steps are taken to ensure data integrity and quality. First, a 50% random subsample of respondents is telephoned after the study visit to assess the overall experience with the interviewers. Issues are reported to EMSI for resolution. Second, all data collection tools are hand-checked for readability, correct skip patterns and missing data as they are received by MSDH. Delta CHES staff call participants to verify information when needed. Lastly, laboratory analyses are conducted using industry recognized standards and are subjected to internal laboratory quality monitoring by the Mississippi Public Health Laboratory and LabCorp.

### 2.8. Data Management

MSDH acts as the data coordinating center for Delta CHES. At the end of the in-home data collection period, MSDH will collate all questionnaires. Data will be entered, processed, and weighed and results will be compiled into one dataset. MSDH logs and ships the FFQs, mailed back by participants, to the Fred Hutchinson Cancer Research Center for scanning and processing. The pedometer diary data are entered into a Delta CHES database as received, and laboratory results are transmitted electronically to MSDH. After data from all sources are received and entered, data will be merged into one master database, matched by the participant’s unique identification number. The final analytic dataset will be in aggregate and void of personal identifiers. All subsequent data analyses will use this dataset to protect the confidentiality of participants.

### 2.9. Notification of Study Findings

A cover letter and a health report summarizing anthropometric measurements and clinically relevant laboratory results are mailed to each participant. Participants are encouraged to share results with their health care provider. A local telephone number is provided in the letter for medical provider referral information for individuals without a health care provider. Participants are called and advised to seek medical care when any laboratory results or blood pressure readings are above predetermined critical values. Participant feedback is based on the urgency for medical attention, using guidelines from the Seventh Report of the Joint National Committee on Prevention, Detection, Evaluation, and Treatment of High Blood Pressure [26].

### 2.10. Incentives

In addition to the health report and pedometer, a $45 gift card is given to each participant as compensation for completing the study visit. Participants receive a $10 gift card for returning the FFQ and a $10 gift card for returning the pedometer diary. The incentives are mailed to the participant’s home together with the cover letter, the health report, and CVD-related educational materials. If the FFQ and/or pedometer daily steps diary are received after the letter and report are mailed, then incentives are mailed immediately following receipt of the FFQ and/or pedometer daily steps diary.

### 2.11. Epidemiological Implications

While data from the national surveillance systems are used to provide reliable, directly measured health data for the nation, comparable state or regional-level data are not available to track leading health indicators and inform local-level policies and interventions. Since data collected by surveillance and monitoring systems allow for the appropriate allocation of limited resources and focused programmatic planning and intervention, localized systems are important.

Delta CHES is an integrated model designed to assess the burden of CVD and examine policy and environmental factors that influence CVD and its risk factors in the 18-county Mississippi Delta region. Delta CHES will fill a gap in applied population health by adding detailed information on chronic disease health outcomes and determinants at the regional level. Some of this information, such as levels of undiagnosed or uncontrolled disease and risk factors, is available for the first time at this level. Data can be used to develop localized CVD control strategies and to support policy and environmental interventions in the Mississippi Delta region that promote health. As the deleterious effects of CVD risk factors occur prior to a diagnosis or adverse health event, the identification and assessment of those unaware of major CVD risk factors, findings unavailable using a telephone-based survey, is a valuable example of one of the benefits of this data collection model. Finally, Delta CHES could serve as a demonstration project on how to successfully recruit, enroll, and collect data, including measured variables, from rural and other difficult to reach populations, and as a model for a future statewide CHES.

## Figures and Tables

**Table 1:** Sociodemographic characteristics of the Mississippi Delta region, Mississippi, and the United States.

Delta county	Population size^a^	% total Delta population	% non-Hispanic black^a^	% non-Hispanic white^a^	% other^a^	Median household income^b^	% persons below FPL^b^	Urban-rural classification^c^,^d^
Bolivar	33,771	6.1	64.2	34.4	1.4	$28,984	33.9	Micropolitan
Carroll	10,373	1.9	33.0	65.5	1.4	$34,436	18.8	Micropolitan
Coahoma	25,913	4.7	75.1	23.7	1.3	$25,719	38.7	Micropolitan
Desoto	164,053	29.6	22.8	74.1	3.1	$58,979	10.4	Large fringe metro
Holmes	18,818	3.4	82.6	16.4	1.0	$22,536	41.2	Noncore
Humphreys	9,312	1.7	74.4	24.3	1.4	$24,205	42.2	Noncore
Issaquena	1,392	0.3	64.2	35.1	0.7	$27,124	43.3	Noncore
Leflore	31,861	5.8	72.5	25.8	1.7	$26,037	37.2	Micropolitan
Panola	34,602	6.2	48.9	49.7	1.4	$32,377	27.9	Noncore
Quitman	8,134	1.5	69.4	29.2	1.4	$25,507	36.9	Noncore
Sharkey	4,892	0.9	70.8	28.3	0.9	$24,987	37.9	Noncore
Sunflower	29,296	5.3	72.4	26.4	1.2	$26,921	39.2	Micropolitan
Tallahatchie	15,318	2.8	56.7	41.1	2.2	$27,352	34.8	Noncore
Tate	28,719	5.2	30.6	67.7	1.7	$41,465	18.2	Large fringe metro
Tunica	10,628	1.9	72.8	25.3	1.9	$29,808	28.1	Large fringe metro
Warren	48,346	8.7	46.9	51.0	2.1	$37,578	23.3	Micropolitan
Washington	50,406	9.1	71.0	27.5	1.5	$25,559	42.2	Micropolitan
Yazoo	27,886	5.0	57.1	41.2	1.7	$28,474	39.0	Micropolitan
Delta Region	553,720		49.7	48.3	2.0	$30,447	25.5	
Mississippi	2,978,512		37.3	60.0	2.6	$36,992	21.7	
United States	311,591,917		13.1	78.1	8.8	$50,046	15.3	

FPL: Federal Poverty Level.

a Data source: U.S. Census Bureau—2011 Census of Population.

b Data source: U.S. Census Small Area Income and Poverty Estimates for State and Counties.

c Data Source: 2006 National Center for Health Statistics Urban-Rural Classification Scheme for Counties.

d Categories and classification rules: large central metro: central counties of MSAs of 1 million or more population; large fringe metro: counties in MSA of 1 million or more population that do not qualify as large central; medium metro: counties within MSAs of 250,000–999,999 population; small metro: counties within MSAS of 50,000 to 249,999 population; micropolitan: counties in micropolitan statistical area; noncore: counties not in micropolitan statistical area [4].

**Table 2:** Age-adjusted death rates for major CVDs, heart disease, and stroke for non-Hispanic blacks and non-Hispanic whites, Mississippi Delta region, Mississippi, and the United States, 2008.

	All			Non-Hispanic black			Non-Hispanic white		
	Major CVDs death rate^a^^b^ (95% CI)	Heart disease death rate^a^^c^ (95% CI)	Stroke death rate^a^^d^ (95% CI)	Major CVDs death rate^a^^b^ (95% CI)	Heart disease death rate^a^^c^ (95% CI)	Stroke death rate^a^^d^ (95% CI)	Major CVDs death rate^a^^b^ (95% CI)	Heart disease death rate^a^^c^ (95% CI)	Stroke death rate^a^^d^ (95% CI)
Delta Region^e^	369.0 (352.3–385.6)	279.6 (265.1–294.0)	56.2 (49.7–62.7)	443.8 (414.4–473.2)	311.0 (286.4–335.5)	79.1 (66.7–91.5)	314.7 (295.0–334.3)	256.8 (239.0–274.5)	40.6 (33.6–47.7)
Mississippi	338.9 (332.3–345.4)	264.3 (258.5–270.1)	52.7 (50.1–55.3)	419.1 (404.7–433.4)	308.6 (296.3–320.9)	74.8 (68.7–80.8)	310.7 (303.3–318.1)	249.5 (242.8–256.1)	44.2 (41.4–47.0)
United States	246.8 (246.2–247.3)	189.0 (188.6–189.5)	41.3 (41.1–41.5)	338.9 (336.7–341.1)	250.2 (248.3–252.1)	60.3 (59.4–61.2)	245.4 (244.8–246.0)	189.9 (189.3–190.4)	39.9 (39.6–40.1)

CVD: cardiovascular disease. Data source: Compressed Mortality File, CDC Wonder (http://wonder.cdc.gov/).

a Rates are per 100,000 population, age-adjusted to the 2000 US standard population.

b ICD-10 codes: I00–I78.

c ICD-10 codes: I00–I09, I11, I13, and I20–I51.

d ICD 10 codes: I60–I69.

e Counties include Bolivar, Carroll, Coahoma, Desoto, Holmes, Humphreys, Issaquena, Leflore, Panola, Quitman, Sharkey, Sunflower, Tallahatchie, Tate, Tunica, Warren, Washington, and Yazoo.

**Table 3:** Power to detect statistically significant differences in rates of diabetes, dyslipidemia, and hypertension between non-Hispanic black and non-Hispanic white residents of the Mississippi Delta region.

Condition	Non-Hispanic blacks Prevalence (%)^a^	Non-Hispanic whites Prevalence (%)^a^	Power (1 – β) for alpha = 0.05	Total sample size
Diabetes	16.0	10.2	0.80	1,006
Dyslipidemia	35.9	45.2	0.80	874
Hypertension	42.5	34.7	0.80	1,234

a From the 2007 and 2009 combined Mississippi Behavioral Risk Factor Surveillance System.

**Table 4:** Mississippi Delta Cardiovascular Health Examination Survey questionnaire domains, physical examination measures, and blood tests.

Category	
Questionnaire domains	Alcohol consumption; anxiety and depression; aspirin use; cholesterol; diabetes; diet and nutrition; general health and access to care; health insurance; hypertension; knowledge of signs and symptoms of heart attack and stroke; medical conditions and family medical history; community perception and environment; occupation; oral health; perceived stress; physical activity and physical fitness; reactions to race; social and emotional support; sociodemographic information and housing; tobacco use and exposure; vitamins and medications; weight history

Physical examination measures	Hip and waist circumference; blood pressure; height; pulse; weight

Blood tests	Complete blood count; comprehensive metabolic panel (alanine aminotransferase; albumin : globulin ratio; albumin, serum; alkaline phosphatase, serum; aspartate aminotransferase; bilirubin, total; bilirubin : creatinine ratio; calcium, serum; carbon dioxide, total; chloride, serum; creatinine, serum; globulin, total; glucose, serum; potassium, serum; protein, total, serum; sodium, serum); insulin; hemoglobin A1c; high sensitivity C-reactive protein; homocysteine; lipid profile (triglycerides, total cholesterol, low-density lipoprotein, high-density lipoprotein); nicotine and metabolite

**Table 5:** Mississippi Delta Cardiovascular Health Examination Survey specimen collection and processing.

Tube number	Tube type	Spin time (minutes)	Transfer	Ship	Ship temperature	Laboratory	Test
1	SST Gold top (5 mL)	15	None	Serum	Ambient	MPHL	Lipid profile
2	EDTA Lavender top (4 mL)	15	Plasma to 1 transfer tube	Plasma	Cold	LabCorp	Homocysteine
3 and 4	EDTA Lavender top (4 mL)	No spin	None	Whole blood	Cold	LabCorp	Hemoglobin A1c; complete blood count
5	Red top (10 mL)	15	Serum to 2 transfer tubes	Serum	Cold	LabCorp	Insulin; nicotine and metabolite
6	SST red/gray top (8.5 mL)	15	None	Serum	Cold	LabCorp	Metabolic panel; high sensitivity CRP

CRP: C-reactive protein; MPHL: Mississippi Public Health Laboratory; SST: Serum Separator Tube.
